# Highly Sensitive and Selective SnO_2_-Gr Sensor Photoactivated for Detection of Low NO_2_ Concentrations at Room Temperature

**DOI:** 10.3390/nano14241994

**Published:** 2024-12-12

**Authors:** Isabel Sayago, Carlos Sánchez-Vicente, José Pedro Santos

**Affiliations:** Institute for Physical and Information Technologies (ITEFI-CSIC), 28006 Madrid, Spain; c.sanchez.vicente@csic.es (C.S.-V.); jp.santos@csic.es (J.P.S.)

**Keywords:** gas sensor, semiconductor oxide, nanoparticles, pollutant gases, NO_2_, graphene, photoactivation, gas mixtures (CO + NO_2_, CH_4_ + NO_2_)

## Abstract

Chemical nanosensors based on nanoparticles of tin dioxide and graphene-decorated tin dioxide were developed and characterized to detect low NO_2_ concentrations. Sensitive layers were prepared by the drop casting method. SEM/EDX analyses have been used to investigate the surface morphology and the elemental composition of the sensors. Photoactivation of the sensors allowed for detecting ultra-low NO_2_ concentrations (100 ppb) at room temperature. The sensors showed very good sensitivity and selectivity to NO_2_ with low cross-responses to the other pollutant gases tested (CO and CH_4_). The effect of humidity and the presence of graphene on sensor response were studied. Comparative studies revealed that graphene incorporation improved sensor performance. Detections in complex atmosphere (CO + NO_2_ or CH_4_ + NO_2_, in humid air) confirmed the high selectivity of the graphene sensor in near-real conditions. Thus, the responses were of 600%, 657% and 540% to NO_2_ (0.5 ppm), NO_2_ (0.5 ppm) + CO (5 ppm) and NO_2_ (0.5 ppm) + CH_4_ (10 ppm), respectively. In addition, the detection mechanisms were discussed and the possible redox equations that can change the sensor conductance were also considered.

## 1. Introduction

One of the pollutants of greatest concern is NO_2_ due to the unprecedented increase it has experienced in recent decades. NO_2_ is generated when fossil fuels such as coal, oil, gas or diesel are burned at high temperatures. NO_2_ causes a variety of adverse health effects and contributes to the development of asthma, respiratory infections and chronic lung diseases. In addition, it is a risk to the environment as it promotes the formation of acid rain, ozone and a pollution cloud (smoky fog) [1].

Nanostructured materials have shown great potential to be used as sensing layers due to their large surface-to-volume ratio, high specific surface area and more active surface site [2]. SnO_2_ is the most widely used material as a resistive-type gas sensor due to its high sensitivity and good stability [3]. Currently, one of the main objectives in the sensor research area is to develop reliable, high-performance gas sensors that can operate at room temperature (RT) [4]. Traditional resistive gas sensors typically have a wide operating temperature range, from 100 to 400 °C. High-temperature operation can reduce the stability and lifetime of the sensor, as well as the high power consumption involved [5]. However, sensors operating at RT do not require a heating element. This leads to simplified manufacturing processes and corresponding cost reductions.

One of the main strategies to develop RT resistive sensors based on semiconductor oxides is to illuminate the sensitive layer with ultraviolet light (UV). Ultraviolet illumination with light energy comparable to or higher than the bandgap of the semiconductor can enhance the conductivity of the material by increasing the number of free carriers. SnO_2_ has a wide bandgap of 3.5 eV; therefore. it can only be activated by light with higher photon energy, i.e., ultraviolet light [6]. In addition, photoactivation of the material can improve the performance of the sensors by increasing their sensitivity and improving the detection processes (faster adsorption/desorption) [7].

Recent studies have revealed that the incorporation of noble metals and/or nanomaterials improves the response at room temperature of photoactivated SnO_2_ sensors. In this sense, Hyodo et al. [8,9] reported that Pd or Pt nanoparticles enhanced NO_2_-sensing properties of thick film SnO_2_-based sensors at RT. Similar results were obtained by J. Wang et al. [10] by incorporating Au into SnO_2_ nanowires. Furthermore, to improve the performance of the photoactivated sensor, the formation of heterojunctions in the sensor material has been proposed, which leads to suppression of the photoexcited recombination of electrons and holes [11].

Currently, graphene and its derivatives have attracted interest due to its unique mechanical and electrical (high electrical conductivity, high carrier mobility and excellent electron transfer rate) properties, high specific surface area and thermal stability [12,13]. SnO_2_-rGO composites have been used for NO_2_ detection at low operating temperatures (120 and 75 °C) [14,15]. However, few articles report on photoactivated sensors based on SnO_2_/graphene composites that work at RT [16,17].

The development of Internet of Things (IoT) technology has led to an emerging demand in the gas sensor market for miniaturized, low-cost sensors that can be incorporated into portable devices and smart mobile terminals to improve quality of life and/or health [18,19].

In this sense, the present work proposes a simple, economical, versatile and potentially scalable method to prepare low-cost sensors by drop casting using automated equipment. In addition, low-cost microsubstrates (FR-4) with very good properties (electrical insulation, mechanical strength and moisture resistance) suitable to be used in the field of gas sensors are employed. Consequently, more versatile and economical sensors could be developed based on more cost-effective and feasible technologies (on an industrial scale).

Here, we present the responses to different pollutant gases (NO_2_, CO and CH_4_) obtained with two low-cost resistive-type sensors. The sensors were based on SnO_2_ nanoparticles (SnO_2_ sensor), and one of them was decorated with graphene (Gr). The detections were carried out in controlled atmospheres of dry and humid air at RT and under UV-illumination of the sensors. The results revealed that the graphene decorated sensor exhibited better sensing performances than the undecorated sensor. The effect of UV-illumination and the humidity presence on the detection processes were discussed. In addition, the SnO_2_-Gr sensor was exposed to complex atmospheres where two pollutant gases were present simultaneously.

## 2. Materials and Methods

Tin oxide nanoparticles (<100 nm particle size), graphene dispersion (1 mg mL^−1^ in DMF) and deionized (DI) water, purchased from Sigma Aldrich (Madrid/Spain), were used. The graphene dispersion contains graphene sheets a few micrometers in size and 1–3 layers thick; and it has an oxygen content of 7.5% and a C/CO:12.3 ratio [20].

The sensitive layers were obtained by drop casting nanoparticle dispersions on FR-4 substrates. The FR-4 substrates were manufactured by Eurocircuits NV (Mechelen, Belgium). FR-4 is a glass-reinforced epoxy laminate material used for printed circuit boards because of its flame resistance, almost zero water absorption and wide operating temperature range (from 50 °C to 115 °C) [21]. On the substrate surface, interdigitated electrodes (IDT) are arranged to allow measuring of the resistance of the sensors. The IDTs consist of four pairs of fingers with a finger width and spacing of 0.23 mm and 0.1 mm, respectively. The active area of the sensor is 2.87 × 2.87 mm^2^.

An automatic “dropcaster” device was used for preparing the different sensitive films. The device characteristics and operating conditions are described in the previous works [22,23]. The device allows for controlling the process parameters such as drop size, deposition time, waiting time between each drop and deposition volume.

### 2.1. Sensor Preparation

Initially, dispersions of SnO_2_ nanoparticles in deionized water were prepared by sonication (2.5 mg L^−1^). The sensitive layers were obtained by drop casting nanoparticle (NP) dispersions on FR-4 substrates. During deposition, the substrate was illuminated by an infrared LED array to promote the solvent evaporation. The 9-LED array used (ILR-IO09-85SL-SC211-WIR200, Osram, Munich, Germany) has an IR centroid wavelength of 850 nm and a maximum radiant intensity of 12,060 mW. The light intensity emitted by the LED array was altered by changing the applied voltage. During deposition, a voltage of 12.5 V was applied, corresponding to a luminous intensity of approximately 1624 mW/cm^2^. A thermographic camera was used to determine the surface temperature of the substrate, which is maintained during the deposition process in the range of 80–85 °C.

The sensors were prepared in the same conditions. Thus, for preparing each sensitive layer, 14 µL of dispersion was used and added to the substrate drop by drop. Each droplet volume was 200 nL, and the waiting time between droplets was 120 s. The graphene-decorated samples were obtained by incorporating a certain amount of the graphene dispersion (200 ppm %wt) into the SnO_2_-NPs dispersions.

SEM/EDX and TEM analyses were used to investigate the surface morphology, and the elemental composition of the sensors. SEM requires little or no sample preparation. However, TEM samples must be mounted onto a TEM grid, and they must be thin enough for electrons to travel through them (ideally 100 nanometers or less).

### 2.2. Experimental Setup

Sensing properties of the sensors were evaluated by an automated multichannel test system. The sensors were mounted in a standard TO-8 package on a printed circuit board for electrical characterization. The whole set was introduced into the stainless steel test cell (0.85 cm^3^) with a gas inlet/outlet.

In order to photoactivate the sensors, a UV-LED (OCU–400 UB355, λ = 353–360 nm, radiant intensity = 0.45 mW, OSA Opto Light GmbH, Berlin, Germany) was integrated into the test cell at 4 mm of sensor so that all the light fell on it in a controlled and uniform way, with reduced power consumption. The experimental setup and schematic of the device were shown in our previous reports [23].

Detections were performed at room temperature both in dry and humid air (50% relative humidity—RH) with a constant gas flow of 100 mL·min^−1^. The exposure time to the target gas was 15 min, while the recovery time was 30 min. The reference gases used were synthetic air (99.999% purity), CH_4_ (10 ppm, diluted in air), CO (10 ppm, diluted in air) and NO_2_ (1 ppm, diluted in air), purchased from Nippon Gases España, S.L.U.

A gas mixing unit (GMU, Ray IE, Cáceres, Spain) with four mass flow meters (one for each reference gas) was used to control the gas mixture concentration and ensure uniform mixing of the gases. An automated gas line allows us to control the mass flow controllers and to adjust the detection parameters such as exposure time and concentration of the gases in the mixtures.

An available humidity generator generates and regulates the relative humidity by controlling the flow of dry and humid air mixtures. This process is managed by a PID controller, which uses real-time readings from humidity and temperature sensors to precisely control the flow rates of both dry and humid air, ensuring the desired gas mixture is achieved. Both types, humidity and temperature sensors, are located in the pre-chamber where the different gases are mixed before being introduced into the cell for measurement.

The flows of the reference gases are established by the following mass balance equation:

c_i_ × f_i_ = f_s_ × c_s_

where c_i_ is the concentration of the reference gases, c_s_ is the desired concentration, f_i_ is the flow rate of the reference gas, and f_s_ is the total gas flow in the test cell (in our case 100 mL min^−1^).

The reference gases were diluted using synthetic air by adjusting the respective flow rates through mass flow controllers while maintaining the total constant flow rate (100 mL min^−1^).

The response of the sensors to target gases was estimated by the resistance change, as follows:

Reducing gases   Response (%) = (R_a_ − R_g_)/R_a_ × 100

Oxidizing gases   Response (%) = (R_g_ − R_a_)/R_g_ × 100

where R_a_ is the initial resistance of the sensor in air atmosphere, and R_g_ is the resistance measured after being exposed to test gas.

The electrical resistance of the sensors was measured with an electrometer (6517 model, Keithley, Cleveland, OH, USA). The processes and equipment were controlled through a PC, using a home-developed software with LabVIEW 2024 Q1 that monitors and displays the resistance of the sensors as a function of time and gas concentration.

## 3. Results and Discussion

### 3.1. Sensitive Layer Characterization

Scanning electron microscopy (SEM) and energy dispersive spectroscopy (EDX, Quanta 3d FEG, FEI company, Hillsboro, OR, USA) were used to study the morphology and composition of the sensitive layer. These were prepared under the same conditions as the sensors but on silicon substrates.

**Figure 1:**
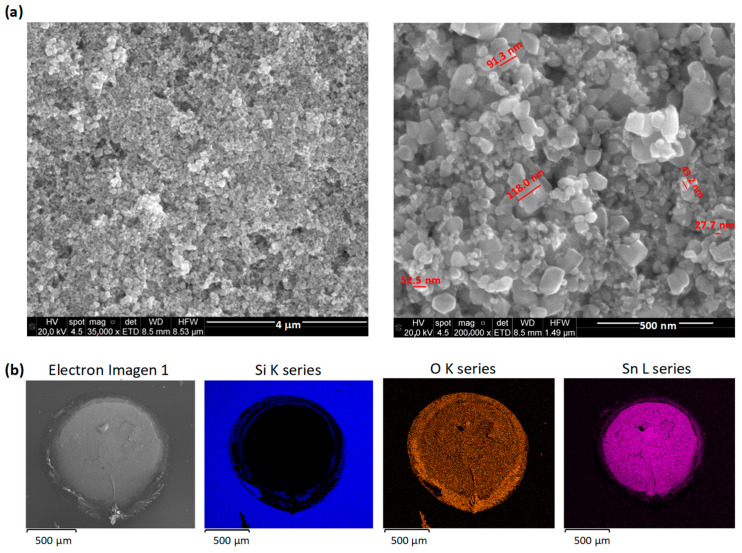
(**a**) SEM micrographs and (**b**) EDX elemental mapping images of one sensitive layer (SnO_2_-Gr).

SEM images show the typical surface morphology, very similar in all samples, with nanoparticle aggregates and nanopores randomly distributed (Figure 1a). The nanoparticles are quasi-spherical with an average size of about 100 nm. Some other NP geometries (rectangular, radial and triangular) are also observed with sizes of the order of 200 nm. Aggregates formation is due to two fundamental causes: on the one hand, the large surface area of the NPs favors the affinity of the nanoparticles among themselves, and on the other hand, the preparation process (drop casting) leads to superposition of the nanoparticles. Both are involved in the formation of randomly distributed asymmetric agglomerates. Finally, we have not observed the presence of graphene on the surface, possibly due to the small amount incorporated in the dispersions (200 ppm). EDX spectra indicated the presence of Sn and O as major elements, and mapping revealed (Figure 1b) that they were uniformly distributed on the surface of the sample.

**Figure 2:**
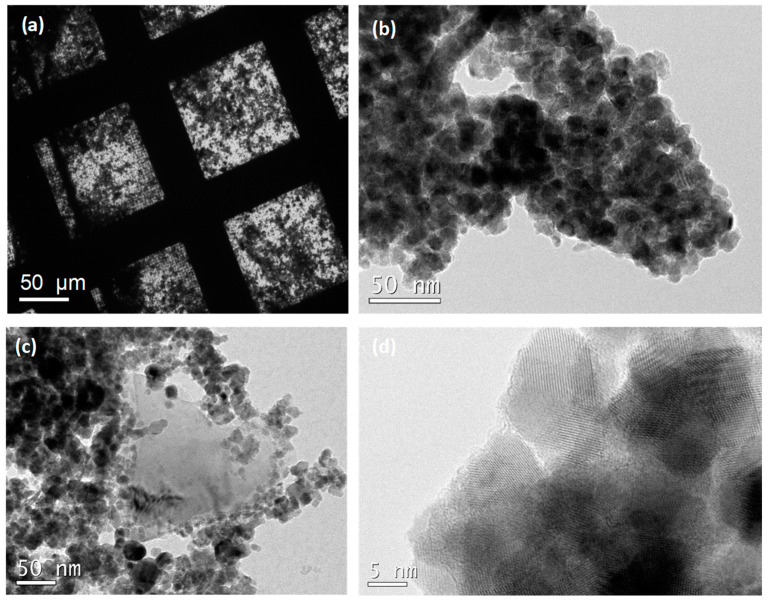
TEM images of (**a**) Gr-SnO_2_ on grids (**b**) pristine SnO_2_ nanoparticles, (**c**) Gr-SnO_2_ and (**d**) HRTEM images of Gr-SnO_2_.

TEM analysis enabled us to observe the nanoparticles in more detail. For this purpose, several drops of the nanoparticle dispersion were deposited on TEM grids. Graphene could not be observed in the samples prepared from SnO_2_-NP dispersions with 200 ppm graphene, so samples were prepared from dispersions with higher amounts of graphene (400, 600 and 1000 ppm %wt). TEM images display the presence of graphene just in the samples with a higher amount of graphene (1000 ppm). TEM micrographs show how nanoparticles and graphene are randomly distributed over the TEM grids (Figure 2a). TEM images show how the SnO_2_ nanoparticles have a spherical morphology and a diameter close to 20 nm, and the NPs tend to form almost spherical agglomerates (Figure 2b,c). Graphene is not visualized on the NPs, and very few individual graphene sheets close to the particles could be observed (Figure 2c). It should be noted that the lattice stripes of the SnO_2_ crystallite are visible in the high-resolution TEM image (Figure 2d).

### 3.2. Sensitive Layer Electrical Characterization

**Figure 3:**
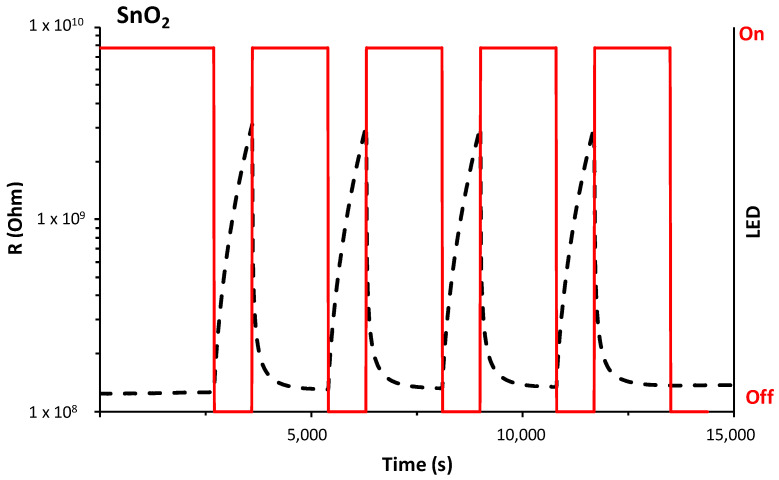
Resistance changes in the SnO_2_-NPs sensor tested with and without UV-LED illumination at RT in air.

The UV-LED illumination of the sensors produces abrupt resistance changes in the tested sensors, as shown in Figure 3 for one of the sensors. In the dark, the sensors have a higher resistance (in the order of 10^9^ and 10^8^ ohms for SnO_2_ and SnO_2_-Gr sensors, respectively). The high surface area of the sensor material (NP) and its small size favor the adsorption of oxygen species on its surface and, consequently, high resistance values. The illumination generates electron–hole pairs and rapid changes in charge carriers (photogenerated electrons). Several investigations have indicated that the photoconductivity of semiconductors is significantly affected by the presence of oxygen in the surrounding atmosphere [23,24,25,26]. When illuminating the semiconductor, photoexcited charge carriers are generated, but the possible interaction of photogenerated charge carriers with molecules adsorbed on the surface of a semiconducting oxide, in particular with chemisorbed oxygen molecules, must also be considered. Therefore, during on/off cycles the sensor resistance variation can be attributed to both photoexcited charge carriers and oxygen photodesorption. As can be seen in Figure 3, the photoactivation of the sensor is fast and requires from 2 to 3 min for the sensor resistance to reach a stable value. 

### 3.3. Gas Sensing Properties

To study the effect of UV-LED illumination on the detection process, the sensors were exposed to different concentrations of target gas (NO_2_, CO, CH_4_) with and without illumination. At RT and in the dark, the sensors only exhibited resistance changes in the presence of NO_2_, although the changes were irreversible (i.e., the initial resistance value was not recovered). However, the sensors showed no resistance changes with the other gases tested (CO and CH_4_).

#### 3.3.1. Sensor Response to NO_2_

**Figure 4:**
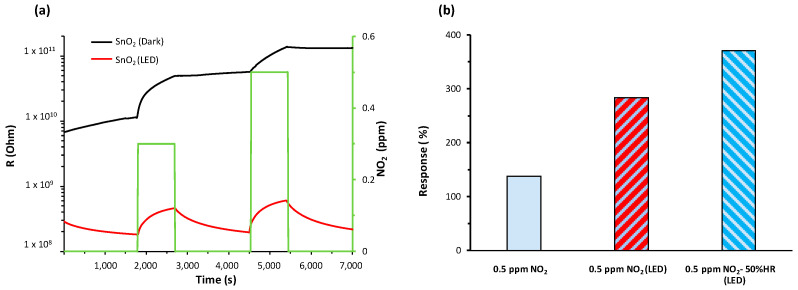
SnO_2_ sensor: (**a**) Dynamic response to NO_2_ at RT in air atmosphere with and without UV-LED illumination and (**b**) responses to 0.5 ppm NO_2_ under different conditions (with and without UV-LED, dry and humid air).

At RT, it is worth noting that the sensors exhibited a decrease in resistance in the presence of NO_2_ regardless of the UV state (Figure 4a), confirming the semiconducting n-type behavior of the sensing layers. Figure 4a shows the detection curves of a SnO_2_ sensor with and without illumination. The illumination improved the performance of the sensor, with a better performance in terms of response (137 vs. 283% without illumination, Figure 4b) and faster and more reversible detection processes (Figure 4a). In addition, humidity increased the response of the sensor from 283% to 372% (Figure 4b).

Ambient humidity is an important factor affecting the performance of the sensors. The results presented here are focused on the detection of the gases tested in the presence of humidity (50% relative humidity). In addition, the sensors were illuminated with a UV-LED during the detection process, as mentioned above.

**Figure 5:**
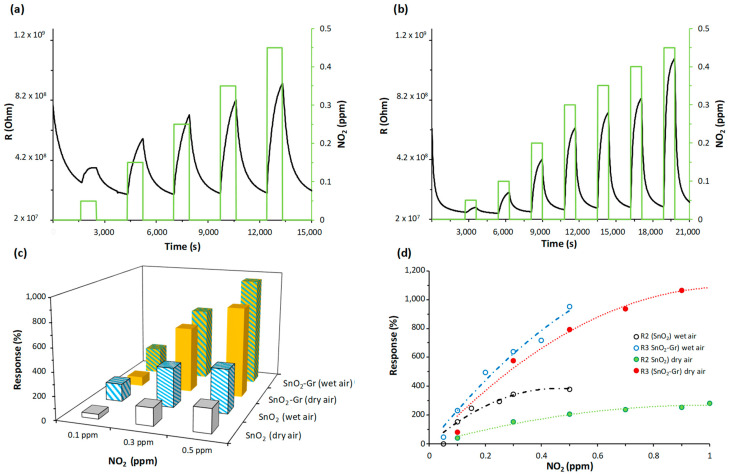
Dynamic response curves at RT under UV-LED illumination to NO_2_ different concentrations of the tested sensors: (**a**) SnO_2_ and (**b**) SnO_2_-Gr. (**c**) Response of the SnO_2_ and SnO_2_-Gr sensors to 0.1, 0.3 and 0.5 ppm NO_2_ at RT under UV-LED illumination in dry and humid air (50% RH). (**d**) Sensor responses versus NO_2_ gas concentration in dry and humid air (50% RH) with UV-LED illumination, where the circles denote experimental results and the dotted lines represent fitting curves.

The sensors were exposed to low concentration ranges of NO_2_ (0.05–0.5 ppm). The experimental detection curves show that the resistance of the sensors gradually increased with NO_2_ concentration (Figure 5a,b). However, the SnO_2_ sensor does not significantly change its resistance in the detection range from 0.25 to 0.45 ppm when compared to the Gr-SnO_2_ sensor, as can be seen in the graph. In humid air, for the SnO_2_ sensor, a tendency toward saturation is observed, perhaps due to the occupation of the adsorption points by chemisorbed hydroxyl groups of water. Considering the sensitivity values, the changes obtained in the SnO_2_ sensor when going from detecting 0.25 ppm NO_2_ to detecting 0.5 ppm NO_2_ are 1.6 times higher in dry air (going from 118% to 188%) and this increase is 1.3 times higher in wet air (going from 293% to 380%). These increases are considerable, although they are not visible in the graph.

The detection processes were reversible and the sensor returned to their initial resistance after several detection cycles, indicating good repeatability. Sensors were tested for two months, and during that period, the operating time was about 400 h. Every period of sensor continuous operation oscillated between 5 and 7 h, and during that time, the resistance values with respect to time and surrounding atmosphere were automatically registered in intervals of 30 s. Additionally, detection cycles were repeated at least twice. The sensor exhibited a good repeatability, obtaining similar sensitivity values for the same detected concentrations.

During the exposure time to NO_2_, the SnO_2_ sensor (Figure 5a) does not reach saturation (its resistance increases), while the Gr-decorated sensor (Figure 5b) has a tendency to saturate (constant resistance). Under these conditions, we have defined the response time of the sensors as the time required to reach a 90% variation in the initial resistance. While the recovery time has been considered the time required for the sensor resistance to recover 70% of its initial value (baseline), both sensors have fast response times, less than one minute. However, the recovery times are higher for SnO_2_ and SnO_2_-Gr sensors, 6 and 3 min, respectively.

The graphene-decorated sensor has a higher response to NO_2_ than the SnO_2_ sensor (Figure 5c,d). In dry air, its response was twice as high as the SnO_2_ response, so for 0.1 and 0.3 ppm, the response was 40% and 152% for the SnO_2_ sensor and 80% and 303% for the SnO_2_-Gr sensor, respectively. However, for concentrations of 0.5 ppm, the increase was considerably higher (almost four times more), with a response of 205% for the SnO_2_ sensor and 793% for the SnO_2_-Gr one. The presence of humidity increases the response of both sensors to NO_2_, so when the concentration was 0.1 ppm and 0.3 ppm, the responses were 154% and 343% for the SnO_2_-Gr sensor and 230% and 640% for the SnO_2_ one, respectively.

The incorporation of graphene in the sensing film reduces the sensor resistance (Figure 5a,b). Graphene contributes electrons to SnO_2_, increasing the charge carriers and, therefore, the conductance of the semiconductor [27]. In addition, it improves the sensitive properties of the sensor, increasing the responses and decreasing the adsorption and desorption times in the detection process (Figure 5).

The dependence of the sensor response to NO_2_ concentration in the different atmospheres (dry/wet) is shown in Figure 5d. The experimental data are fitted to second degree polynomial functions with regression coefficients higher than 0.97 for the tested sensors except for SnO_2_, which, in the presence of humidity, is 0.91. As mentioned above, in dry air atmosphere, the response of SnO_2_-Gr sensors is twice as high for concentrations below 0.3 ppm and almost four times higher for concentrations above 0.3 ppm. The presence of humidity increases the response of the sensors, but in the case of Gr-decorated sensors, this increase is slightly smaller. At concentrations above 0.3 ppm, the SnO_2_ sensor does not exhibit significant differences in its response and tends to a nearly constant value regardless of the concentration detected (Figure 5d) when humidity is present.

The Materials. To evaluate the sensitive properties of the tested sensors, we compared our results to those reported in the literature references (Table 1). Table 1 summarizes the main NO_2_-sensitive properties of resistive sensors based on different materials and nanostructures. References on the state-of-the-art sensors operating at moderate temperatures (T ≤ 200 °C) and detecting low NO_2_ concentrations (≤1 ppm) have been selected. Ultraviolet light illumination or the incorporation of graphene are commonly used alternatives to reduce the operating temperature of the devices, improving their performance [6,12,13,28]. Thus, ZnO nanomaterial sensors of various morphologies such as nanospheres or light-activated nanowires can detect at RT [29,30,31,32]. Additionally, sensors based on heterojunctions between semiconductors [33,34] or semiconductor and graphene [14,16,35,36] allow operation at moderate temperatures and even at RT without illumination.

All the sensors presented in Table 1 detect low concentrations and the response/recovery times reported are fast. Although NO_2_ concentrations are a few ppms, the values detected by most sensors are higher than those reported in this work. Only the WS_2_ sensor (2D materials) detects 0.1 ppm NO_2_ at RT even without photoactivation [37]. However, the WS_2_ sensor presents a lower response than our sensors, and no data on response/recovery times are available. In addition, the preparation of the sensors involved long, complex and expensive processes that are difficult to incorporate into the production chain for manufacturing low-cost sensors. Recently, we have reported that low-cost sensors based on ZnO nanoparticles decorated with terbium and UV illuminated showed good performances to NO_2_ at RT in dry and humid environments [23]. These sensors were also prepared by drop casting in a similar way to those presented in this work; however, they have lower responses than those obtained by the tin oxide NP sensors reported here.

#### 3.3.2. Selectivity

Selectivity is a critical indicator to establish the performance of the sensor and its viability for marketing and use in real-world conditions (when multiple gases and aromas are present in the environment). Selectivity is the ability of a sensor to respond to a certain gas, regardless of the presence of other species. If the presence of a gas other than the target gas affects the performance of the sensor, this is referred to as an interference. Ideally, a sensor should respond to a single gas, i.e., without interferences. However, gas sensors are usually sensitive to more than one gas and exhibit cross-sensitivity [27].

Selectivity can be defined as the relative response of a sensor to two different analytes. In this work, we have considered percent selectivity as the following ratio: maximum response of the interfering gas to the maximum response of the target gas [39,40].

(% Selectivity) = (Response_other gas_/Response_Target gas_) × 100%.

**Figure 6:**
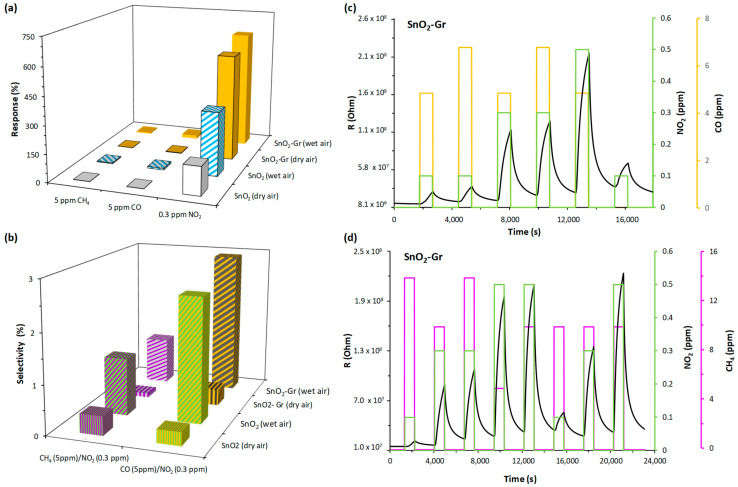
(**a**) Responses of the sensors to 0.3 ppm NO_2_, 5 ppm CO and 5 ppm CH_4_ in dry and wet air. (**b**) Selectivity of the tested sensors to NO_2_ at RT and under UV-LED illumination, in dry and humid air (50%). SnO_2_-Gr sensor dynamic response at RT in humid air (45% RH) and under UV-LED illumination to different gas mixtures: (**c**) mixture 1 (NO_2_ + CO) (**d**) mixture 2 (NO_2_ + CH_4_).

To study the selectivity of the sensors, they were exposed to other contaminant gases such as CO and CH_4_. Figure 6a shows the responses of the sensors to the different detected gases NO_2_ (0.3 ppm), CO (5 ppm) and CH_4_ (5 ppm) in both atmospheres tested (dry and wet). The sensors exhibited a greater response to CO than to CH_4_. Thus in humid atmosphere, for 5 ppm CO, the response of the SnO_2_-Gr sensor was 18.73%, twice the SnO_2_ sensor response (8.7%), while the responses at 5 ppm CH_4_ of the SnO_2_-Gr and SnO_2_ sensors were 6% and 4%, respectively. The sensors exhibited a very high response to NO_2_ (Figure 6a). Thus, the responses in the wet environment of SnO_2_ and SnO_2_-Gr sensors to 0.3 ppm NO_2_ were ~104 and ~85 times higher, respectively, than the response to 5 ppm CH_4_ and 39 and 34 times higher than the response to 5 ppm CO. Figure 6b illustrates, in both dry and humid air, the selectivity to NO_2_ of the tested sensors with respect to each secondary gas considered (CO and CH_4_). The sensors presented an excellent selectivity to NO_2_ with practically no cross-responses to interfering gases (R_CH4_ or _CO_/R_NO2_ ~0), in dry air and low cross-response to both gases (R_CH4_/R_NO2_ < 1.2 and R_CO_/R_NO2_ < 3) in humid air.

#### 3.3.3. Detection in Complex Atmospheres

The results previously presented allowed us to evaluate the sensitivity and selectivity of the sensors. Considering the excellent sensing properties of the tested sensors, in particular the SnO_2_-Gr sensor, which was selected to study its behavior in complex atmospheres (air, water vapor and two atmospheric pollutants), these detections approximate the real operating conditions for the sensors.

Most of the reported studies on resistive-type gas sensors are mainly focused on the investigation of single gases, and few of them report detections in complex atmospheres [41,42]. In recent years, due to the poor selectivity of the resistive sensors, the possibility of developing multimodal sensors with several detection mechanisms [43,44] has been investigated capable of estimating the compositions of gas mixtures in a selective and quantitative manner. However, it is necessary to know properly the behavior of the sensors in a controlled atmosphere in order to select the optimal sensors according to the detection gas.

Due to the measurement system characteristics (constant total gas flow of 100 mL/m and humid air generated by mixing dry and humid air), the humidity has been limited to 45% to widen the range of gas concentrations present in the mixture. Here, we have evaluated the SnO_2_-Gr sensor response in gas mixtures (mixture 1 and 2, Table 2) consisting of humid air (45% RH) and two gases, one oxidizing (NO_2_) and the other reducing (CO or CH_4_).

Figure 6c,d show the detection curves of the SnO_2_-Gr sensor for the different mixtures detected at RT with 45% RH and UV-LED illumination. The experimental curves illustrate that the sensor increases its resistance (oxidizing effect) in the presence of the gas mixtures formed by an oxidizing gas (NO_2_) and a reducing gas (CO or CH_4_). Moreover, the sequence of the responses matches the order observed in the NO_2_ detection tests (Table 2), implying that the sensor responds to NO_2_ despite the higher concentration of the reducing gases. These results confirm the selectivity of the sensor to NO_2_, as established in the previous section. The sensitivity and selectivity of the sensor are higher to NO_2_ than to the tested reducing gases (CO and CH_4_); this is due to NO_2_ peculiarities. High electronic affinity and paramagnetism (i.e., an unpaired electro) NO_2_ gas complement each other, facilitating the adsorption on the semiconductor. It can therefore be adsorbed in multiple equations, as is discussed below (section on detection mechanisms). This facilitates the adsorption of NO_2_ on the active points of the sensor and, therefore, resulting in a much higher affinity compared to the other non-magnetic gases tested (CO and CH_4_).

### 3.4. Detection Mechanism

Detection mechanisms of resistive sensors activated by illumination can be interpreted similarly to the hypotheses formulated in dark detection, i.e., they are due to the adsorption/desorption process of oxygen on the material surface of the sensing materials. In the dark at temperatures below 150 °C, with an atmosphere where oxygen is present as in the air case, oxygen is adsorbed as O_2_^−^ [45,46,47,48].

In the dark, the responses of our sensors are characterized by slow and irreversible sensing processes, whose results seemed to indicate that the most probable interaction type is chemisorption. In contrast, sensing processes under illumination with fast response and recovery times suggested that the sensing mechanism is due to weak interaction between the detected gas and active sites on the semiconductor surface.

The sensor illumination involves two main steps [49,50,51]: (1) Photon adsorption with an energy higher than the bandgap; (2) Generation, separation, migration or recombination of photogenerated electron–hole pairs.

**Figure 7:**
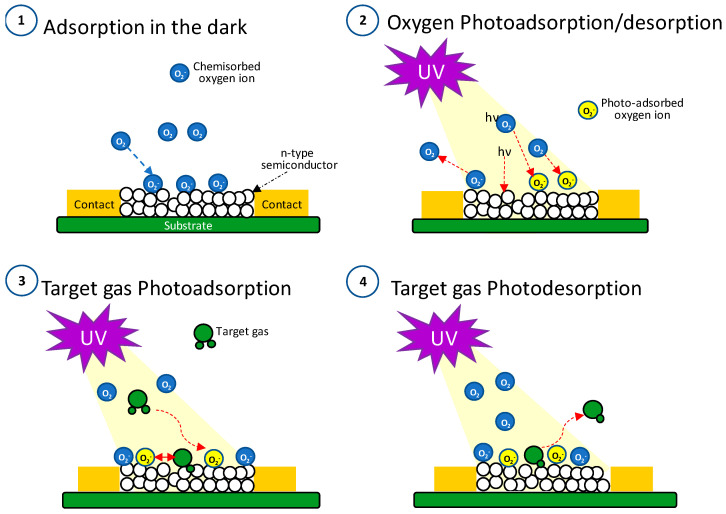
Detection mechanism scheme.

Figure 7 illustrates the interaction mechanism that we consider appropriate to describe the sensor response based on models found in the literature [46,47,48,49,52], correlated to our experimental results. In addition, we have considered the possible equations involved in the sensing process.

Step 1, in the dark, the oxygen adsorption occurs [45,46,47,48], Equation (1):

O_2_(g) + e^−^ → O_2_^−^_(ads)_ (1)

Step 2, under illumination, according to the report of Fan et al. [52], the photoinduced electrons diffuse into the semiconductor (increasing the conductivity of the sensing material), Equation (2):

hν _(UV)_ → e^−^_(hν)_ + h^+^_(hν)_ (2)

while the photoinduced holes migrate to the surface and can react with the adsorbed oxygen species (O_2_^−^_ads_), producing the oxygen desorption on the surface, and therefore, sensor resistance decreases [53,54,55], Equation (3):

Desorption O_2_^−^_(ads)_ + h^+^_(hν)_ → O_2_ (3)

Simultaneously, the photoinduced electrons can interact with the oxygen molecules of the air, producing photoinduced oxygen species (O_2_^−^_(hυ)_), Equation (4):

Adsorption O_2_(g) + e^−^_(hν)_ → O_2_^−^_(hν)_ (4)

According to reports in the literature, the phothoinduced species are weakly bound to the sensor surface [51,52]. 

Step 3, photoadsorption of the target gas. According to our experimental results, we consider the prevailing and determining mechanism is due to photoactivated oxygen species (O_2_^−^_(hυ)_). Moreover, considering that light-generated species are generally weakly bound to the material surface [56,57,58], the adsorption/desorption processes of the different species can occur rapidly and reversibly even at RT. Therefore, it improves the gas detection efficiency that matches our experimental data.

Step 4, photodesorption of the target gas.

Depending on the gas detected, electrons will be injected or extracted from the SnO_2_ conduction band, causing an increase or decrease in the sensor resistance. The possible equations that take place with the detected gases:

(a) In the case of NO_2_ (oxidizing gas), as it occurs in the dark, it interacts with the semiconductor surface by direct adsorption, Equation (5):
  NO_2_ + e^−^_(hν)_/e^−^ → NO_2_^−^_(ads)_ (5)
  Moreover, since its electronic affinity is higher than the O_2_ respective one [59], it can extract electrons from the adsorbed oxygen species, Equation (6). In both cases, the electron capture occurs, possibly photogenerated electrons, increasing the sensor resistance [60,61,62].
  NO_2_ + O_2_^−^_(hν)_ + 2e^−^_(hν)_/e^−^ → NO_2_^−^_(ads)_/NO_3_^−^_(ads)_ + 2O^−^ (6)
  Our sensors have high responses to NO_2_, and we consider that Equations (5) and (6) were carried out simultaneously.
(b) In the case of reducing gases, the oxidation equation was carried out, producing CO_2_ [61,63], Equations (7) and (8):
  CO + O_2_^−^_(hν)_ → CO_2_ + e^−^ (7)
  CH_4_ + O_2_^−^_(hν)_ → CO_2_ + 2H_2_O + 2e^−^ (8)
(c) In the detection of mixtures containing NO_2_ and a reducing gas (CO or CH_4_), competitive equations could occur between the adsorbed oxygen species and the gases tested. NO_2_, due to its electronic affinity, attracts electrons easily and adsorption Equations (5) and (6) can occur simultaneously during the detection process. As a result, the active sites on the sensor surface will be preferentially occupied by NO_2_ adsorbed species, blocking the adsorption of the reducing gases. This can explain the excellent selectivity of the tested sensors to NO_2_.

The gas detection equations take place on the sensing material surface. The high response of the tested sensors can be attributed to the nanoparticle size. Nanoparticles with a high surface area favor the adsorption of gas molecules on their surface, which leads to a higher sensor response.

As previously discussed, the improved sensing properties of the SnO_2_/graphene-based sensor can be attributed to the electronic interaction between graphene and SnO_2_ nanoparticles. Due to the formation of p-n heterojunctions in the interface between SnO_2_ (n-type semiconductor) and graphene (p-type semiconductor), electrons can be transferred from SnO_2_ to graphene, since n-type semiconductors have an excess of electrons, while p-type semiconductors contain an excess of holes. In addition, graphene sheets with a high surface area provide active sites for gas adsorption, but they can also prevent the agglomeration of nanoparticles, increasing the active sites of SnO_2_ for gas adsorption [64]. As a result, the number of adsorbed molecules is higher, and conductivity changes occur more rapidly, improving the performance of the sensor.

The presence of humidity involves the dissociation of water molecules and the adsorption of OH^−^ and H^+^ ions on the sensor surface [65,66]. Photoactivation of OH^−^ groups can generate OH* radicals, according to the following equation [67,68]:

OH^−^_(ads)_ + h^+^_(hυ)_ → OH*_(ads)_ (9)

Studies by Nasriddinov et al. about the surface of SnO_2_ confirmed that in a humid atmosphere, O_2_^−^ species can be easily replaced by OH* radicals [69]. The OH^*^ radical is a strong oxidant and it could interact with carbon-containing gases, such as CH_4_ or CO [67,68], releasing electrons and producing a sensor resistance decrease. This would justify the increased response to reducing gases observed in our sensors in humid atmosphere.

In the case of NO_2_, the interactions are diverse and complex and generally involve the formation of nitrate as an intermediate species [70,71]. On one hand, hydroxyl groups (OH^−^) are active sites for the adsorption of NO_2_ molecules, with formation of hydrogen bonds between the OH^−^ and the NO_2_ molecule [72]. Moreover, the hydroxyl radical could also interact with the NO_2_ molecule [73]. It should not be forgotten that direct adsorption can also occur, Equation (5). In any case, it is possible that all these interactions could also occur simultaneously, which would imply an increase in the response to NO_2_ that would be in agreement with the results observed in our sensors.

## 4. Conclusions

In this work, we develop low-cost sensors for NO_2_ detection based on SnO_2_ nanoparticles and SnO_2_ nanoparticles decorated with graphene. Sensor responses to NO_2_ and their selectivity to other pollutant gases are investigated. The performances of sensors were established according to their response, response/recovery time to NO_2_ and their cross-sensitivity against CO and CH_4_ (reducing gases).

Photoactivation of the sensitive layers improved the performance of sensors at RT; the response to NO_2_ was doubled and it allowed the detection of reducing gases that were not detected in the dark. In addition, illumination activated the detection processes, reducing the response and recovery times.

The addition of graphene to tin oxide improved the conductance of the sensor, increased the response to NO_2_, accelerated the sensing process (reaching saturation during the 15 min exposure time) and decreased the recovery time. So, the SnO_2_ sensor response to 0.3 ppm NO_2_ increased from 152.9% to 575% by incorporating graphene. Both sensors, decorated and non-decorated ones, presented fast response times, less than 1 min. However, the recovery times were longer, 6 min for SnO_2_ sensor and 3 min for the graphene-decorated sensor.

Humidity has a positive effect on sensor responses, probably due to the accumulation of hydroxyl groups and hydroxyl radicals on the sensor surface that act as active sites for adsorption of the target gas. Hydroxyl radicals were probably generated due to UV-illumination.

The sensors showed high sensitivity and selectivity to NO_2_, detecting concentrations as low as 0.1 ppm. Thus, the SnO_2_ sensor response in humid air to 0.3 ppm NO_2_ was 86 times greater than the response to CH_4_ (5 ppm) and 18 times greater than the response to CO (5 ppm). Regarding the SnO_2_-Gr sensor, the responses were superior: 104 times higher than to CH_4_ and 34.1 times higher than to CO. In addition, a low cross-response to the interferential gases CO and CH_4_ was obtained with a R_CH4_ or _CO_/R_NO2_ ratio close to zero for both sensors.

Detections in complex atmosphere (CO + NO_2_ or CH_4_ + NO_2_ in humid air) confirmed the selectivity of the graphene sensor in near-real conditions. Their responses were of the same order of magnitude regardless of the interfering gas presence. So, they were of 600% to 0.5 ppm NO_2_, 657% to NO_2_ (5 ppm) +CO (5 ppm) and 540% to NO_2_ (5 ppm) + CH_4_ (10 ppm).

The developed approach can be used for preparing high-performance gas-sensing devices that are more versatile and economical than the current ones. On one hand, economical microsubstrates of FR-4 (glass-reinforced epoxy laminate material), a common material for printed circuit boards and other electronic components, are used. In addition, these substrates present very good properties for their application in the field of gas sensors since they allow the incorporation of associated electronics for controlling and recording measurements and they facilitate the development of portable sensor devices. On the other hand, the easily scalable drop-casting method will permit the preparation of nanomaterial sensing films in very small areas, facilitating a mass production. Consequently, more versatile and cost-effective sensors could be developed based on more cost-effective and feasible (industrial scale) technologies.

## Figures and Tables

**Table 1:** Sensing properties of resistive sensors at low NO_2_ concentrations.

Material/Morphology	Conc. (ppm)	Working Temp. (°C)	Response *	Response/Recovery Time (s)	Ref.
ZnO/Nanosheet	0.5	200	76 ^b^	-/-	[29]
ZnO/nanospheres	0.5	RT-UV	2.2 ^b^	12 s/35 s	[30]
ZnO/nanoflowers	0.25	200	11 ^b^	-/-	[31]
ZnO Nanowires	1	RT-UV	780 ^a^	31 s/144 s	[32]
ZnO-rGO	0.1	130	6.35 ^b^	17 s/16–66 s	[35]
ZnO-Tb/NPs	0.1	RT-UV	54 ^a^ (wet air)	120 s/360 s	[23]
WS_2_/Nanosheets	0.1	RT	9.3 ^a^	-/-	[37]
SnO_2_@ZnO	0.2	RT-UV	4.17 ^a^	<60 s	[33]
SnO-SnO_2_/p-n	0.2	RT	2.5 ^b^	57 s/300 s	[34]
SnO_2_/microspheres	1	RT	2.36 ^b^	Few seconds	[38]
SnO_2_-rGO	0.5	120	84.5 ^a^	22 s/125 s	[14]
rGO/SnO_2_	1	60	13 ^a^	-/-	[36]
SnO_2_-graphene	0.5	RT-UV	7.5 ^a^	-/10 s	[16]
SnO_2_/NPs	0.1	RT-UV	40 ^a^/154 ^a^ (dry air/wet)	<60 s/360 s	This work
SnO_2_-Gr/NPs	0.1	RT-UV	80 ^a^/230 ^a^ (dry air/wet)	<60 s/180 s	This work

* Note, a: S (%) =  (Ra − Rg)/Ra × 100; b: S =  (Rg/Ra).

**Table 2:** SnO_2_-Gr sensor response (%) to gas mixture (M1 and M2) at RT with 45% RH under UV-LED illumination.

Response (%) Mixture 1	Mixture 1 (NO_2_ + CO)		Response (%) Mixture 2	Mixture 2 (NO_2_ + CH_4_)	
	NO_2_ (ppm)	CO (ppm)		NO_2_ (ppm)	CH_4_ (ppm)
125.4	0.1	7	88.2	0.1	10
404.4	0.3	7	385.3	0.3	10
657.6	0.5	5	593.6	0.5	10
106.9	0.1	0			
422.2	0.3	0	419.7	0.3	10
606.7	0.5	0	540.8	0.5	10

## Data Availability

Data are contained within the article.

## References

[1] https://www.eea.europa.eu/publications/zero-pollution/health/air-pollution.

[2] Dey A. (2018). Semiconductor metal oxide gas sensors: A review. Mater. Sci. Eng. B.

[3] Kang X., Deng N., Yan Z., Pan Y., Sun W., Zhang Y. (2022). Resistive-type VOCs and pollution gases sensor based on SnO_2_: A review. Mater. Sci. Semicond. Process..

[4] Wang Z., Bu M., Hu N., Zhao L. (2023). An overview on room-temperature chemiresistor gas sensors based on 2D materials: Research status and challenge. Compos. Part. B Eng..

[5] Mirzaei A., Lee J., Majhi S.M., Weber M., Bechelany M., Kim H.W., Kim S.S. (2019). High-temperature operation can reduce the stability and lifetime of the sensor as well as the high-power consumption involved. J. Appl. Phys..

[6] Sun C., Yang J., Xu M., Cui Y., Ren W., Zhang J., Zhao H., Liang B. (2022). Recent intensification strategies of SnO_2_-based photocatalysts: A review. J. Chem. Eng..

[7] Law M., Kind H., Messer B., Kim F., Yang P. (2002). Photochemical Sensing of NO_2_ with SnO_2_ Nanoribbon Nanosensors at Room Temperature. Angew. Chem. Int. Ed..

[8] Hyodo T., Urata K., Kamada K., Ueda T., Shimizu Y. (2017). Semiconductor-type SnO_2_-based NO_2_ sensors operated at room temperature under UV-light irradiation. Sens. Actuators B Chem..

[9] Saboor F.H., Ueda T., Kamada K., Hyodo T., Mortazavi Y., Khodadadi A.A., Shimizu Y. (2016). Enhanced NO_2_ gas sensing performance of bare and Pd-loaded SnO_2_ thick film sensors under UV-light irradiation at room temperature. Sens. Actuators B Chem..

[10] Zhang B., Zhang S., Xia Y., Yu P., Xu Y., Dong Y., Wei Q., Wang J. (2022). High-Performance Room-Temperature NO_2_ Gas Sensor Based on Au-Loaded SnO_2_ Nanowires Under UV Light Activation. Nanomaterials.

[11] Zhao L., Chen Y., Li X., Li X., Lin S., Li T., Rumyantseva M.N., Gaskov A.M. (2019). Room temperature formaldehyde sensing of hollow SnO_2_/ZnO heterojunctions under UV-LED activation. IEEE Sens. J..

[12] Wang T., Huang D., Yang Z., Xu S., He G., Li X., Hu N., Yin G., He D., Zhang L. (2016). A Review on Graphene-Based Gas/Vapor Sensors with Unique Properties and Potential Applications. Nano-Micro Lett..

[13] Choi J.H., Lee J., Byeon M., Hong T.E., Park H., Lee C.Y. (2020). Graphene-Based Gas Sensors with High Sensitivity and Minimal Sensor-to-Sensor Variation. ACS Appl. Nano Mater..

[14] Modak M., Jagtap S. (2022). Low temperature operated highly sensitive, selective and stable NO_2_ gas sensors using N-doped SnO_2_-rGO nanohybrids. Ceram. Int..

[15] Wang Z., Wang Z., Jia Z., Li Q., Zhang X., Sun W., Sun J., Liu B., Ha B. (2019). The enhanced NO_2_ sensing properties of SnO_2_ nanoparticles/reduced graphene oxide composite. J. Colloid. Interface Sci..

[16] Zhang Z., Gao Z., Fang R., Li H., He W., Du C. (2020). UV-assisted room temperature NO_2_ sensor using monolayer graphene decorated with SnO_2_ nanoparticles. Ceram. Int..

[17] Pargoletti E., Hossain U.H., Di Bernardo I., Chen H., Tran-Phu T., Chiarello G.L., Lipton-Duffin J., Pifferi V., Tricoli A., Cappelletti G. (2020). Engineering of SnO_2_–Graphene Oxide Nanoheterojunctions for Selective Room-Temperature Chemical Sensing and Optoelectronic Devices. ACS Appl. Mater. Interfaces.

[18] Zhu J., Cho M., Li Y., He T., Ahn J., Park J., Ren T.-L., Lee C., Park I. (2021). Machine learning-enabled textile-based graphene gas sensing with energy harvesting-assisted IoT application. Nano Energy.

[19] Gomes J.B.A., Rodrigues J.J.P.C., Rabêlo R.A.L., Kumar N., Kozlov S. (2019). IoT-Enabled Gas Sensors: Technologies, Applications, and Opportunities. J. Sens. Actuator Netw..

[20] https://www.sigmaaldrich.com/ES/es/product/aldrich/900450.

[21] https://www.protoexpress.com/blog/why-fr4-material-in-pcb-fabrication/.

[22] Santos J., Sanchez-Vicente C., Azabal A., Ruiz-Valdepenas S., Lozano J., Sayago I., Sanjurjo J. Automation and optimization device for the fabrication of sensors with nanomaterials. Proceedings of the 2021 13th Spanish Conference on Electron Devices (CDE).

[23] Sayago I., Santos J.P., Sánchez-Vicente C. (2022). The Effect of Rare Earths on the Response of Photo UV-Activate ZnO Gas Sensors. Sensors.

[24] Cunningham R.D., Marton J.P., Schlesinger M. (1969). Photoconductivity in SnO_2_ Crystals. J. Appl. Phys..

[25] Gurwitz R., Cohen R., Shalish I. (2014). Interaction of light with the ZnO surface: Photon induced oxygen breathing, oxygen vacancies, persistent photoconductivity, and persistent photovoltage. J. Appl. Phys..

[26] Chizhov A., Rumyantseva M., Gaskov A. (2021). Light Activation of Nanocrystalline Metal Oxides for Gas Sensing: Principles, Achievements, Challenges. Nanomaterials.

[27] Pargoletti E., Tricoli A., Pifferi V., Orsini S., Longhi M., Guglielmi V., Cerrato G., Falciola L., Derudi M., Cappelletti G. (2019). An electrochemical outlook upon the gaseous ethanol sensing by Graphene oxide-SnO_2_ hybrid materials. Appl. Surf. Sci..

[28] Xu F., Ho H.-P. (2017). Light-Activated Metal Oxide Gas Sensors: A Review. Micromachines.

[29] Van Duy L., Nguyet T.T., Hung C.M., Le D.T.T., Van Duy N., Hoa N.D., Biasioli F., Tonezzer M., Di Natale C. (2021). Ultrasensitive NO_2_ gas sensing performance of two dimensional ZnO nanomaterials: Nanosheets and nanoplates. Ceram. Int..

[30] Wang H., Dai M., Li Y., Bai J., Liu Y., Li Y., Wang C., Liu F., Lu G. (2021). The influence of different ZnO nanostructures on NO_2_ sensing performance. Sens. Actuators B Chem..

[31] Agarwal S., Rai P., Gatell E.N., Llobet E., Güell F., Kumar M., Awasthi K. (2019). Gas sensing properties of ZnO nanostructures (flowers/rods) synthesized by hydrothermal method. Sens. Actuators B.

[32] Wang J., Shen Y., Li X., Xia Y., Yang C. (2019). Synergistic effects of UV activation and surface oxygen vacancies on the room-temperature NO_2_ gas sensing performance of ZnO nanowires. Sens. Actuators B.

[33] Zhang Z., Xu M., Liu L., Ruan X., Yan J., Zhao W., Yun J., Wang Y., Qin S., Zhang T. (2018). Novel SnO_2_@ZnO hierarchical nanostructures for highly sensitive and selective NO_2_ gas sensing. Sens. Actuators B.

[34] Yu H., Yang T., Wang Z., Li Z., Zhao Q., Zhang M. (2018). p-N heterostructural sensor with SnO-SnO_2_ for fast NO_2_ sensing response properties at room temperature. Sens. Actuators B.

[35] Qu G., Fan G., Zhou M., Rong X., Li T., Zhang R., Sun J., Chen D. (2019). Graphene-Modified ZnO Nanostructures for Low-Temperature NO_2_ Sensing. ACS Omega.

[36] Zhu X., Guo Y., Ren H., Gao C., Zhou Y. (2017). Enhancing the NO_2_ gas sensing properties of rGO/SnO_2_ nanocomposite films by using microporous substrates. Sens. Actuators B.

[37] Xu T., Liu Y., Pei Y., Chen Y., Jiang Z., Shi Z., Xu J., Wu D., Tian Y., Li X. (2018). The ultra-high NO_2_ response of ultra-thin WS_2_ nanosheets synthesized by hydrothermal and calcination processes. Sens. Actuators B.

[38] Zhou L., Hu Z., Li H.-Y., Liu J., Zeng Y., Wang J., Huang Y., Miao L., Zhang G., Huang Y. (2021). Template-Free Construction of Tin Oxide Porous Hollow Microspheres for Room-Temperature Gas Sensors. ACS Appl. Mater. Interfaces.

[39] Aswal D.K., Gupta S.K. (2007). Science and Technology of Chemiresistor Gas Sensors.

[40] Mirzaei A., Lee J.-H., Majhi S.M., Weber M., Bechelany M., Kim H.W., Kim S.S. (2019). Resistive gas sensors based on metal-oxide nanowires featured. J. Appl. Phys..

[41] Patil D.R., Patil L.A. (2007). Room temperature chlorine gas sensing using surface modified ZnO thick film resistors. Sens. Actuators B Chem..

[42] Chu J., Li W., Yang X., Wu Y., Wang D., Yang A., Yuan H., Wang X., Li Y., Rong M. (2021). Identification of gas mixtures via sensor array combining with neural networks. Sens. Actuators B Chem..

[43] Yin J. (2023). Rapid Identification Method for CH_4_/CO/CH_4_-CO Gas Mixtures Based on Electronic Nose. Sensors.

[44] Han H.J., Cho S.H., Han S., Jang J., Lee G.R., Cho E.N., Kim S., Kim I., Jang M.S., Tuller H.L. (2021). Synergistic Integration of Chemo-Resistive and SERS Sensing for Label-Free Multiplex Gas Detection. Adv. Mater..

[45] Raza M., Chen Y., Trapp J., Sun H., Huang X., Ren W. (2023). Smoldering peat fire detection by time-resolved measurements of transient CO_2_ and CH_4_ emissions using a novel dual-gas optical sensor. Fuel.

[46] McAleer J.F., Moseley P.T., Norris J.O.W., Williams D.E. (1987). Tin dioxide gas sensors. Part 1—Aspects of the surface chemistry revealed by electrical conductance variations. J. Chem. Soc. Faraday Trans..

[47] Harrison P.G., Willett M.J. (1989). Tin oxide surfaces. Part 20—Electrical properties of tin(IV) oxide gel: Nature of the surface species controlling the electrical conductance in air as a function of temperature. J. Chem. Soc. Faraday Trans..

[48] Yamazoe N., Fuchigami J., Kishikawa M., Seiyama T. (1979). Interactions of tin oxide surface with O_2_, H_2_O and H_2_. Surf. Sci..

[49] Gurlo A. (2006). Interplay Between O_2_ and SnO_2_: Oxygen Ionosorption and Spectroscopic Evidence for Adsorbed Oxygen. Chem. Phys. Chem..

[50] Soci C., Zhang A., Xiang B., Dayeh S.A., Aplin D.P.R., Park J., Bao X.Y., Lo Y.H., Wang D. (2007). ZnO Nanowire UV Photodetectors with High Internal Gain. Nano Lett..

[51] Park S., Sun G.-J., Jin C., Kim H.W., Lee S., Lee C. (2016). Synergistic Effects of a Combination of Cr_2_O_3_-Functionalization and UV-Irradiation Techniques on the Ethanol Gas Sensing Performance of ZnO Nanorod Gas Sensors. ACS Appl. Mater. Interfaces.

[52] Fan S.-W., Srivastava A.K., Dravid V.P. (2009). UV-activated room-temperature gas sensing mechanism of polycrystalline ZnO. Appl. Phys. Lett..

[53] Lupan O., Cretu V., Postica V., Ahmadi M., Cuenya B.R., Chow L., Tiginyanu I., Viana B., Pauporté T., Adelung R. (2016). Silver-doped zinc oxide single nanowire multifunctional nanosensor with a significant enhancement in response. Sens. Actuators B Chem..

[54] Muraoka Y., Takubo N., Hiroi Z. (2009). Photoinduced conductivity in tin dioxide thin films. J. Appl. Phys..

[55] Espid E., Taghipour F. (2017). UV-LED Photo-Activated Chemical Gas Sensors: A Review. Crit. Rev. Solid State Mater. Sci..

[56] Chopra K., Major S., Pandya D. (1983). Transparent conductors—A status review. Thin Solid Film..

[57] Zhang C., Boudiba A., De Marco P., Snyders R., Olivier M.G., Debliquy M. (2013). Room temperature responses of visible-light illuminated WO_3_ sensors to NO_2_ in sub-ppm range. Sens. Actuators B Chem..

[58] Isimjan T.T., El Ruby A., Rohani S., Ray A.K. (2010). The fabrication of highly ordered and visible-light-responsive Fe-C-N-codoped TiO_2_ nanotubes. Nanotechnology.

[59] Zhang C., Geng X., Li J., Luo Y., Lu P. (2017). Role of oxygen vacancy in tuning of optical, electrical and NO_2_ sensing properties of ZnO_1−x_ coatings at room temperature. Sens. Actuators B Chem..

[60] Linstrom P.J., Mallard W.G. (2013). NIST Standard Reference Database Number 69.

[61] Zhang C., Luo Y., Xu J., Debliquy M. (2019). Room temperature conductive type metal oxide semiconductor gas sensors for NO_2_ detection. Sens. Actuators A Phys..

[62] Gurlo A., Riedel R. (2007). In Situ and Operando Spectroscopy for Assessing Mechanisms of Gas Sensing. Angew. Chem. Int..

[63] Zhang X., Tang Y., Li Y., Wang Y., Liu X., Liu C., Luo S. (2013). Reduced graphene oxide and PbS nanoparticles co-modified TiO_2_ nanotube arrays as a recyclable and stable photocatalyst for efficient degradation of pentachlorophenol. Appl. Catal. A Gen..

[64] Wang C., Wang Y., Yang Z., Hu N. (2021). Review of recent progress on graphene-based composite gas sensors. Ceram. Int..

[65] Kohl D. (1989). Surface processes in the detection of reducing gases with SnO_2_-based devices. Sens. Actuators.

[66] Barsan N., Weimar U. (2001). Conduction Model of Metal Oxide Gas Sensors. J. Electroceram..

[67] Sun S., Ding J., Bao J., Gao C., Qi Z., Li C. (2010). Photocatalytic Oxidation of Gaseous Formaldehyde on TiO_2_: An In Situ DRIFTS Study. Catal. Lett..

[68] Liu L., Li X., Dutta P.K., Wang J. (2013). Room temperature impedance spectroscopy-based sensing of formaldehyde with porous TiO_2_ under UV illumination. Sens. Actuators B Chem..

[69] Nasriddinov A., Rumyantseva M., Konstantinova E., Marikutsa A., Tokarev S., Yaltseva P., Fedorova O., Gaskov A. (2020). Effect of Humidity on Light-Activated NO and NO_2_ Gas Sensing by Hybrid Materials. Nanomaterials.

[70] Kantcheva M.M., Bushev V.P., Hadjiivanov K.I. (1992). Nitrogen dioxide adsorption on deuteroxylated titania (anatase). J. Chem. Soc. Faraday Trans..

[71] Nanayakkara C.E., Larish W.A., Grassian V.H. (2014). Titanium Dioxide Nanoparticle Surface Reactivity with Atmospheric Gases, CO_2_, SO_2_, and NO_2_: Roles of Surface Hydroxyl Groups and Adsorbed Water in the Formation and Stability of Adsorbed Products. J. Phys. Chem. C.

[72] Yang Z., Jiang L., Wang J., Liu F., He J., Liu A., Lv S., You R., Yan X., Sun P. (2021). Flexible resistive NO_2_ gas sensor of three-dimensional crumpled MXene Ti_3_C_2_Tx/ZnO spheres for room temperature application. Sens. Actuators B Chem..

[73] Dalton J., Janes P., Jones N., Nicholson J., Hallam K., Allen G. (2002). Photocatalytic oxidation of NO_x_ gases using TiO_2_: A surface spectroscopic approach. Environ. Pollut..

